# Lewy and his inclusion bodies: Discovery and rejection

**DOI:** 10.1590/1980-57642016dn11-020012

**Published:** 2017

**Authors:** Eliasz Engelhardt, Marleide da Mota Gomes

**Affiliations:** 1 Cognitive and Behavioral Neurology Unit, INDC - CDA-IPUB - UFRJ, Rio de Janeiro RJ - Brazil; 2 Neurologist, Associate Professor, Program of Epilepsy, Institute of Neurology Deolindo Couto, School of Medicine, UFRJ, Rio de Janeiro, RJ - Brazil

**Keywords:** Lewy, inclusion bodies, eosinophilic, Lewy bodies, Paralysis agitans, Parkinson's disease, Lewy, corpos de inclusão, eosinófilos, corpos de Lewy, Paralysis agitans, doença de Parkinson

## Abstract

Fritz Jacob Heinrich Lewy described the pathology of Paralysis agitans [Parkinson disease] and was the first to identify eosinophilic inclusion bodies in neurons of certain brain nuclei, later known as Lewy bodies, the pathological signature of the Lewy body diseases. In 1912, he published his seminal study, followed soon after by an update paper, and 10 years later, in 1923, by his voluminous book, where he exhaustively described the subject. The publication provided extensive information on the pathology of Paralysis agitans, and the entirely novel finding of eosinophilic inclusion bodies, which would become widely recognized and debated in the future. His discovery was acknowledged by important researchers who even named the structure after him. However, after his last publication on the issue, inexplicably, he never mentioned his histopathological discovery again. Despite several hypotheses, the reasons that led him to neglect (reject) the structure which he so preeminently described have remained elusive.

## Abstract (pt)

Fritz Jacob Heinrich Lewy descreveu a patologia da Paralysis agitans [doença de Parkinson] e identificou pela primeira vez corpos de inclusão eosinófílos em neurônios de certos núcleos cerebrais, conhecidos mais tarde como corpos de Lewy, assinatura patológica das doenças dos corpos de Lewy. Ele divulgou em 1912 seu trabalho seminal, seguido logo por um artigo de atualização e 10 anos depois, em 1923, seu volumoso livro onde detalhou exaustivamente o assunto. Ali ele trouxe extensa informação sobre a patologia da Paralysis agitans e um achado inteiramente novo, os corpos de inclusão eosinófilos, que seriam valorizados e largamente debatidos no futuro. Seu achado foi reconhecido por importantes pesquisadores que até designaram essa estrutura com seu nome. Entretanto, após sua última publicação sobre o assunto, inexplicavelmente , ele nunca mais mencionou sua descoberta histopatológica. Apesar de diversas hipóteses, a razão que o levou negligenciar (rejeitar) a estrutura, que teve a primazia de descrever, permaneceu desconhecida.

## INTRODUCTION

The Lewy bodies represent the neuropathological signature of a group of illnesses that constitute the Lewy body diseases, comprising Parkinson disease and Parkinson disease dementia, and Dementia with Lewy bodies.^1^ The name stems from that of the investigator who first described the structure.

Here, details on Lewy and his discovery will be outlined. Intriguingly, despite this devoted work and recognition it received, he appeared to neglect (reject) his finding, seemingly without a clear explanation, the reasons for which remain a subject of debate.

## LEWY

Fritz Jacob Heinrich Lewy (1885-1950) was a German-American neurologist and neuropathologist, born in Berlin (Germany) and deceased in Philadelphia (United States), bearing the name of Frederick Henry Lewey.^2,3^

Lewy's training was honed by distinguished personalities, mainly Oppenheim (neurology), Kraepelin (psychiatry), Alzheimer, Nissl, and Spielmeyer (neuropathology), von Monakow (neuroanatomy), Magnus (neurophysiology), and he pursued his scientific activities in Germany (Munich, Wroclaw [Breslau], Berlin), England (London), and the United States (Philadelphia).^3,4^ His scientific work was carried out across several disciplines, in different institutions, cities and countries, and was conveyed through his over 200 publications.^3,5^ Most notable was his interest in creating the Neurological Institute in Berlin, also with the intention of separating neurology from psychiatry, to which it was subsumed at the time.^3,6^ The project was begun in 1926, and despite strong opposition, the Institute was constructed and finally inaugurated in 1932, headed by Lewy as the Director. However, with the rise of the NS-Regime in 1933, Lewy was soon dismissed.^3,6,7^ In the same year, Lewy fled to England, where he stayed for one year, and from whence he emigrated to the United States, definitively.^3,4^

## LEWY: THE EOSINOPHILIC INCLUSIONS DISCOVERY

A small number of Lewy's numerous publications addressed Paralysis agitans and related subjects.^3,5^ Three of these studies, published in 1912, 1914 and 1923, warrant special comment^8-10^ and are outlined below. In the texts, he provided a detailed histopathological analysis of the different levels of the nervous system to further understanding on the pathological underpinnings of shaking palsy (Paralysis agitans) (Parkinson disease, as named by Charcot and Vulpian, in 1862).^11^

The seminal study was performed during 1910 and 1911, and published in 1912 in Lewandowsky's Handbook of Neurology ("Pathological Anatomy" of Forster and Lewy's chapter on "Paralysis agitans").^3^ All levels of the brain were examined, and there he identified, for the first time, peculiar intraneuronal inclusions. He first described the formations inside neurons of the dorsal nucleus of the vagus, which he thought were more related to the disease: "These changes are characterized as inclusions, which partly in their genesis, apparently have to do with those pictured by Lafora... and by him assigned...to the structure of the *Corpora amylacea*..." [*Amyloidkörper* or *Amyloidkörperchen* (amylaceous bodies or corpuscles), as published in 1911 by Lafora and Glueck^12^]. And added: "...spherical (globular), strandlike, and serpiginous (serpentine) forms are evident that stain bright red with Mann's technique [methyl blue-eosin mixture]". He also identified similar structures in Meynert's nucleus and in the thalamic paraventricular nucleus.^8^

He held an updating conference "On the pathological anatomy of Paralysis agitans" in 1913 before the Association of German Neurologists, published in 1914. Therein, he emphasized the changes in Meynert's nucleus, and reiterated his former findings: "...Mann's stain reveals a plasma density (compaction) with a spherical (globular) or elongated shape, which exhibit some reactions in the *Corpora amylacea*, but can also be stained with eosin". Very similar changes were also described in the nucleus of the vagus, where he added that: "...appears... a very prominent mass that is divided into an external glassy (hyaline) and internal darker region...". Additionally, he reported these structures in extraneuronal localization.^9^

His study was further extended in the massive monography "The Study on Muscle Tone and Movement. Including Systematic Investigations on the Clinic, Physiology, Pathology, and Pathogenesis of Paralysis agitans", published in 1923. This book represented the culmination of his research on Paralysis agitans. He meticulously scrutinized all levels of the brain, with the use of a large repertoire of histological techniques, and expanded the description of the histopathological changes.^13^ The changes found in Meynert's and vagus nuclei were confirmed. He also described progressive stages of degenerative change in the large ganglion cells of Meynert's nucleus which he designated "glassy [hyaline] cell plasma disease" or "glassy cell disease", besides different types of spherical formations (*Corpora amylacea*, plasma bodies, inclusions) (with Mann's stain).^10^

## LEWY: THE REJECTION

The three studies Lewy published on the pathology of Paralysis agitans, between 1912 and 1923, as seen above, represented an exhaustive work, certainly the result of many years of relentless clinical and pathological studies. These carried extensive information on the pathology of Paralysis agitans, and an entirely novel finding, the eosinophilic inclusion bodies, that would become widely recognized and debated in the future (e.g., Kosaka et al., 1984; Hansen et al., 1990; McKeith et al., 1996; Fujishiro et al., 2008). Except for a precis^14^ he published one year after his book, based on its content without presenting new data, he never again mentioned his histopathological finding.^3,5^

It has been argued, to explain this, that his scientific life was marked by frequent changes of disciplines and institutions, cities and countries, interruption by WW I, and difficulties due to the rise of the anti-Semitic NS-government, including the frustration of his removal from the Institute of Neurology while in Germany, precluding him from pursuing this line of research.^3,5^ He also faced many difficulties in his short stay in England, and in the United States, having had to deal with adaptation difficulties and insecurity in relation to his professional activities, besides the interruption due to WW II.^3,15^

It is also plausible that he changed his research direction, engaging in other themes according to new scientific opportunities and interests that arose over time. Indeed, after the book and its resumé, he went on to publish numerous papers - between 1923 and 1933, before he left Germany, he published 82 papers, plus a further 72 papers while in the United States,^3^ demonstrating his high scientific productivity in both periods, with interest in varied themes. Therefore, there was no reason not to mention his finding in the 1942 publication, the "Historical Introduction: the Basal Ganglia and their Diseases", presented in 1940 and published as Chapter I of the Proceedings of the Association for Research in Nervous and Mental Diseases (The Diseases of the Basal Ganglia),^16^ when already established in the United States. In this publication, he addressed the main diseases related to the pathology of the basal ganglia, including Paralysis agitans, summarizing that: "The pathological characteristics of Paralysis agitans consisted of the subcortical localization of senile and presenile processes predominantly in the globus pallidus, basal nucleus and corpus striatum; but the pathological changes were widespread over the whole central nervous system from the cortex to the spinal ganglia, specifically including the vegetative nuclei of hypothalamus, brain stem and medulla oblongata".^16^ He also cited his 1912 and (1913) 1914 publications, but omitted the massive 1923 book, and surprisingly, made no mention of the peculiar inclusions which he had been the first to describe, and that had already been acknowledged by Lafora (1913),^17,18^ Trétiakoff (1919),^19^ Foix (1921),^20^ and and Hassler (1938),^21^ who had even named the finding after him. It is clear that he was aware of investigations on the subject by other researchers, as he wrote: "The work of the French (Tretiakoff, Foix, Souques) and a lecture of Goldstein pointed to very marked changes in the substantia nigra. Tretiakoff found such changes in 9 examined cases, and Souques in 3 cases of typical Paralysis agitans".^10,14^ Thus, he had at his disposal information from the French school and, as he acknowledged, also from German authors, and possibly others.^14^ This was a unique opportunity to allude to his finding, which he apparently disdained.^5^ Why the omission? It is possible that he understood that, in the big picture, his discovery represented but a minor feature, not worthy of mention. Therefore, the possibility of his own misjudgment on the importance of the new histopathological structure cannot be discarded.^5,7^

Thus, despite the hypotheses put forward, the reasons that led him to reject the formation that he had been the first to describe look set to remain elusive.

## CONCLUSION

Lewy described the pathology of Paralysis agitans, identifying a novel structure - eosinophilic inclusion bodies in neurons of some brain nuclei. After his 1912 seminal paper, and another that followed shortly after, he prepared and published, 10 years later, a massive book on the subject. After this, he never returned to the theme, nor mentioned his original finding. Despite several hypotheses, the reasons that led him to neglect (reject) the structure he so preeminently described, appear set to remain unsolved.
